# Association of L-type amino acid transporter 1 (LAT1) with the immune system and prognosis in invasive breast cancer

**DOI:** 10.1038/s41598-022-06615-8

**Published:** 2022-02-17

**Authors:** Sasagu Kurozumi, Kyoichi Kaira, Hiroshi Matsumoto, Masafumi Kurosumi, Takehiko Yokobori, Yoshikatsu Kanai, Chikako Sekine, Chikako Honda, Ayaka Katayama, Mio Furuya, Sho Shiino, Takaya Makiguchi, Nigel P. Mongan, Emad A. Rakha, Tetsunari Oyama, Takaaki Fujii, Ken Shirabe, Jun Horiguchi

**Affiliations:** 1 Department of Breast Surgery, International University of Health and Welfare, 852, Hatakeda, Narita, Chiba 286-8520 Japan; 2 Department of General Surgical Science, Gunma University Graduate School of Medicine, Gunma, Japan; 3 Department of Respiratory Medicine, Comprehensive Cancer Center, International Medical Center, Saitama Medical University, Saitama, Japan; 4 Division of Breast Surgery, Saitama Cancer Center, Saitama, Japan; 5 Department of Pathology, Saitama Cancer Center, Saitama, Japan; 6 Gunma University Initiative for Advanced Research (GIAR), Maebashi, Gunma Japan; 7 Division of Bio-System Pharmacology, Graduate School of Medicine, Osaka University, Osaka, Japan; 8 Department of Diagnostic Pathology, Gunma University Graduate School of Medicine, Gunma, Japan; 9 Nottingham Breast Cancer Research Centre, School of Medicine, University of Nottingham, Nottingham, UK; 10 Department of Oral and Maxillofacial Surgery and Plastic Surgery, Gunma University Graduate School of Medicine, Gunma, Japan; 11 Biodiscovery Institute, Faculty of Medicine and Health Sciences, University of Nottingham, Nottingham, UK

**Keywords:** Breast cancer, Prognostic markers

## Abstract

L-type amino acid transporter 1 (LAT1), also referred to as SLC7A5, is believed to regulate tumor metabolism and be associated with tumor proliferation. In invasive breast cancer, we clinicopathologically investigated the utility of LAT1 expression. LAT1 expression was evaluated via immunohistochemistry analyses in 250 breast cancer patients undergoing long-term follow-up. We assessed the relationships between LAT1 expression and patient outcomes and clinicopathological factors. Breast cancer-specific survival stratified by LAT1 expression was assessed. Human epidermal growth factor receptor 2 (HER2)-positive patients with metastasis received trastuzumab therapy. The density of tumor-infiltrating lymphocytes (TILs) was evaluated according to the International Working Group guidelines. In the current study, high LAT1 expression was significantly correlated with estrogen receptor (ER) negativity, progesterone receptor negativity, high histological grade, increased TILs, and programmed death ligand 1 positivity. Among the ER-positive and HER2-negative patients, high LAT1 was an independent indicator of poor outcomes (hazard ratio (HR) = 2.97; 95% confidence interval (CI), 1.16–7.62; *p* = 0.023). Moreover, high LAT1 expression was an independent poor prognostic factor in luminal B-like breast cancer with aggressive features (HR = 3.39; 95% CI 1.35–8.52; *p* = 0.0094). In conclusion, high LAT1 expression could be used to identify a subgroup of invasive breast cancer characterized by aggressive behavior and high tumor immunoreaction. Our findings suggest that LAT1 might be a candidate therapeutic target for breast cancer patients, particularly those with luminal B-like type breast cancer.

## Introduction

The survival of patients with breast cancer (BCa) has been improved by recent advances in treatment. However, approximately 20% of BCa patients have a poor prognosis with recurrence and metastasis^1^. Characterizing the factors associated with tumor progression may lead to the identification of new molecular therapeutic targets. Uncontrolled proliferation alters the metabolism and progression of BCa cells and depends on the uptake of sugars and amino acids^2^. Amino acids, including glutamine, are known to play a particularly important role in cell proliferation via the mTOR pathway^3^. The L-type amino acid transporter (LAT) enables the transport of large neutral essential amino acids into cells^4,5^. Interestingly, LAT1, encoded by the *SLC7A5* gene, is generally overexpressed in malignant cells^6–15^. High LAT1 expression is closely related to the proliferation of tumor cells and angiogenesis in various types of cancer, such as melanoma^16^, lung cancer^17^, pancreatic cancer^18^, gastrointestinal cancer^19,20^, and triple-negative BCa (TNBC)^21^. LAT1 overexpression is also associated with lymphovascular invasion, lymphatic metastasis, and advanced stages of cancer^22^ and contributes to the development of therapeutic resistance in cancer cells. A previous large BCa study by El Ansari et al.^23^ reported that a high LAT1 expression level was associated with high proliferation potential, as indicated by the high Nottingham prognostic index and Ki67 labeling index; moreover, high LAT1 expression level was a poor prognostic factor in luminal B-like type breast tumors.

The immune system affects all phases of tumor growth from initiation to progression and dissemination. In addition, previous studies confirmed that tumor immunity-associated biomarkers, such as tumor-infiltrating lymphocytes (TILs) and programmed death ligand 1 (PD-L1), are related to the treatment response and prognosis of BCa^24–27^. Recently, El Ansari et al.^28^ reported that LAT1 expression was associated with the expressions of PD-L1, PD1, FOXP3, CD68 and CD20 in the breast tumor microenvironment.

In this study, we attempted to validate the relationship between LAT1 expression and patient outcome. We also confirmed the correlation between LAT1 protein expression and key clinicopathological factors, including TILs and PD-L1, in BCa patients.

## Results

### Association of LAT1 with clinicopathological factors

High LAT1 expression was present in 124 BCa patients (49.6%), whereas 126 patients (50.4%) had low LAT1 expression. Across the entire cohort, high LAT1 expression was significantly correlated with estrogen receptor (ER) negativity (*p* < 0.0001), progesterone receptor (PgR) negativity (*p* < 0.0001), human epidermal growth factor receptor 2 (HER2) positivity (*p* < 0.0001), large tumor size (*p* = 0.016), and high histological grade (*p* < 0.0001) (Table 1). In The Cancer Genome Atlas (TCGA) cohort, *SLC7A5* mRNA overexpression was associated with ER negativity (*p* < 0.0001), PgR negativity (*p* < 0.0001), large tumor size (*p* = 0.047), high histological grade (*p* < 0.0001), high *Myc* mRNA expression (*p* < 0.0001), high *VEGFA* mRNA expression (*p* < 0.0001) and high *VEGFC* (*p* < 0.0001) mRNA expression (Supplementary Table 1).

**Table 1:** Association of LAT1 expression with clinicopathological factors in all patients.

Factors	Expression of LAT1			Significance
	Low	High	Total	*p*-value
**ER**				
Positive	115 (68.0%)	54 (32.0%)	169	< 0.0001
Negative	11 (13.6%)	70 (86.4%)	81	
**PgR**				
Positive	98 (67.6%)	47 (32.4%)	145	< 0.0001
Negative	28 (26.7%)	77 (73.3%)	105	
**HER2**				
Positive	9 (20.9%)	34 (79.1%)	43	< 0.0001
Negative	117 (56.5%)	90 (43.5%)	207	
**Tumor size**				
pT2-4	51 (42.1%)	70 (57.9%)	121	0.016
pT1	75 (58.1%)	54 (41.9%)	129	
**Nodal status**				
Positive	55 (48.7%)	58 (51.3%)	113	0.70
Negative	71 (51.8%)	66 (48.2%)	137	
**Histological grade**				
Grade 3	47 (32.4%)	98 (67.6%)	145	< 0.0001
Grade 1, 2	79 (75.2%)	26 (24.8%)	105	
**TILs**				
High	6 (19.4%)	25 (80.6%)	31	< 0.0001
Intermediate	14 (34.1%)	27 (65.9%)	41	
Low	106 (59.6%)	72 (40.4%)	178	
**PD-L1**				
Positive	2 (10.0%)	18 (90.0%)	20	0.00024
Negative	122 (53.5%)	106 (46.5%)	228	

*LAT1* L-type amino acid transporter 1, *ER* estrogen receptor, *PgR* progesterone receptor, *HER2* human epidermal growth factor receptor 2, *TIL* tumor-infiltrating lymphocytes.

For the relationships between LAT1 expression and tumor immunity-related biomarkers, high LAT1 expression was significantly associated with high TILs (*p* < 0.0001) and PD-L1 positivity (*p* = 0.00024) (Table 1). Twenty-five (20.2%) patients with high LAT1 were included in the high TIL group, and 18 (14.5%) patients with high LAT1 had PD-L1 positivity. Survival curves stratified by LAT1/TIL and LAT1/PD-L1 levels are shown in Supplementary Fig. 1.

### Outcome analyses

**Figure 1:**
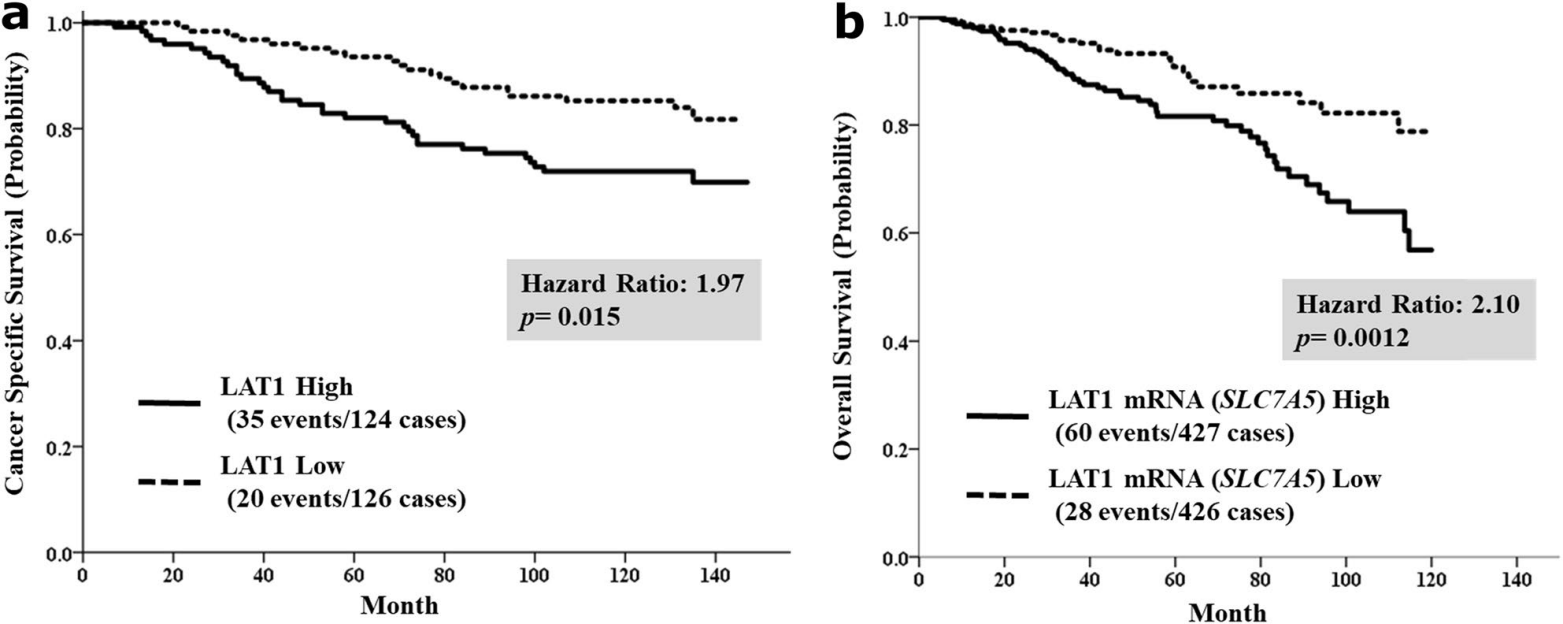
(**a**) Breast cancer-specific survival stratified by L-type amino acid transporter 1 (LAT1) protein expression, (**b**) overall survival stratified by LAT1 mRNA expression.

BCa-specific survival (BCSS) differed significantly between the high and low LAT1 expression groups in the univariable analyses (hazard ratio (HR) = 1.97; 95% confidence interval (CI), 1.14–3.42; *p* = 0.015; Fig. 1a). Univariable analysis also identified large tumor size (HR = 2.05; 95% CI 1.18–3.55; *p* = 0.011), positive nodal status (HR = 3.80; 95% CI 1.74–2.44; *p* < 0.0001), negative ER status (HR = 2.13; 95% CI 1.26–3.62; *p* = 0.0051) and negative PgR status (HR = 2.34; 95% CI 1.36–4.01; *p* = 0.0021) as poor prognostic factors. Positive nodal status was an independent poor prognostic factor in multivariable analysis (HR = 3.47; 95% CI 1.88–6.39; *p* < 0.0001; Supplementary Table 2). The prognosis of the high *SLC7A5* mRNA expression group was significantly worse than that of the low *SLC7A5* mRNA expression group in the TCGA cohort (HR = 2.10; 95% CI 1.34–3.29; *p* = 0.0012; Fig. 1b).

### Prognostic analysis of *SLC7A5* mRNA based on ER status

To obtain insight into how LAT1 might be linked to survival, we evaluated the prognostic utility of *SLC7A5* mRNA expression according to ER status using the TCGA database. Among patients with ER-positive cancer, those with high *SLC7A5*-expressing tumors had a significantly lower BCSS than those with low *SLC7A5*-expressing tumors (HR = 2.13; 95% CI 1.22–3.71; *p* = 0.0075). In contrast, *SLC7A5* expression was not a prognostic factor among patients with ER-negative cancer (Supplementary Fig. 2). At the protein level, high LAT1 expression was a significant prognostic marker in the ER-positive type (HR = 2.22; 95% CI 1.07–4.59; *p* = 0.032) but not in the ER-negative type (Supplementary Fig. 2). Moreover, LAT1 expression was not a significant prognostic marker in the HER2-positive type (Supplementary Fig. 3).

### Prognostic utility of LAT1 expression in ER-positive and HER2-negative BCa

**Figure 2:**
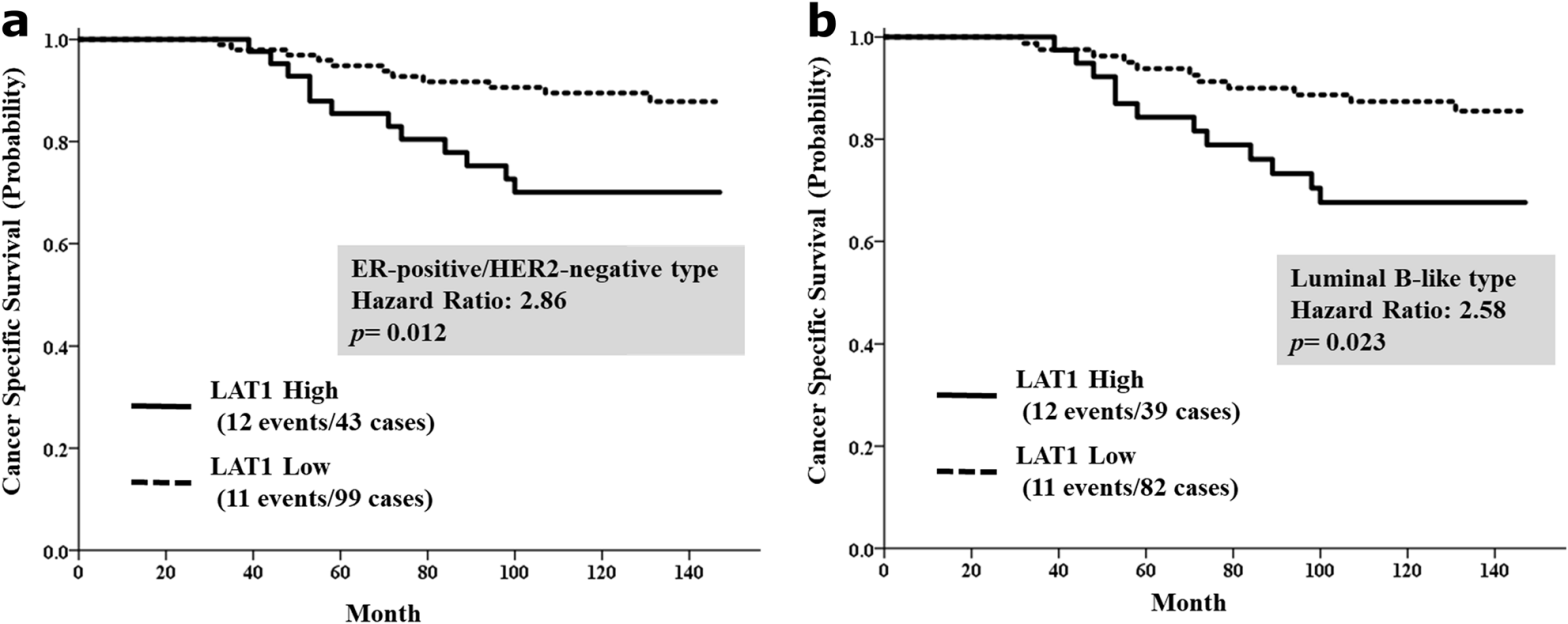
Breast cancer-specific survival stratified by L-type amino acid transporter 1 (LAT1) expression (**a**) in ER-positive and HER2-negative patients and (**b**) in luminal B-like patients.

In the 142 ER-positive and HER2-negative tumors, we investigated which factors were associated with LAT1 staining. The frequency of high LAT1 expression was 39.4% in luminal-like tumors. Among luminal-like tumors, high LAT1 expression was significantly correlated with tumor size (*p* = 0.018), high Ki67 labeling index (*p* = 0.0017), and high histological grade (*p* = 0.018) (Table 2). In patients with luminal-like cancer, those with high LAT1-expressing tumors had a significantly lower BCSS than those with low LAT1-expressing tumors (HR = 2.86; 95% CI 1.26–6.48; *p* = 0.012; Fig. 2a).

**Table 2:** Relationship between LAT1 expression and clinicopathological factors in ER-positive/HER2-negative breast cancer.

Factors	Expression of LAT1			Significance
	Low	High	Total	*p*-value
**Ki67**				
≥ 30%	14 (46.7%)	16 (53.3%)	30	0.0017
> 10 and < 30%	56 (70.9%)	23 (29.1%)	79	
≤ 10%	29 (87.9%)	4 (12.1%)	33	
**PgR**				
Positive	64 (71.9%)	25 (28.1%)	89	0.57
Negative	35 (66.0%)	18 (34.0%)	53	
**Tumor size**				
pT2-4	36 (59.0%)	25 (41.0%)	61	0.018
pT1	63 (77.8%)	18 (22.2%)	81	
**Nodal status**				
Positive	46 (74.2%)	16 (25.8%)	62	0.36
Negative	53 (66.3%)	27 (33.8%)	80	
**Histological grade**				
Grade 3	38 (59.4%)	26 (40.6%)	64	0.018
Grade 1, 2	61 (78.2%)	17 (21.8%)	78	

*LAT1* L-type amino acid transporter 1, *ER* estrogen receptor, *PgR* progesterone receptor, *HER2* human epidermal growth factor receptor 2.

In addition to high LAT1 expression, univariable analysis showed that negative PgR expression (HR = 5.37; 95% CI 2.12–13.63; *p* = 0.00041) and positive nodal status (HR = 4.12; 95% CI 1.64–10.57; *p* = 0.0027) were predictive of reduced survival (Table 3). Multivariable analysis with a Cox proportional hazards regression model identified that LAT1 expression was an independent poor prognostic factor in patients with ER-positive and HER2-negative BCa (HR = 2.97; 95% CI 1.16–7.62; *p* = 0.023; Table 3). Moreover, among luminal B-like type cancer (the aggressive phenotype), high LAT1 expression was an independent poor prognostic factor in univariable (HR = 2.58; 95% CI 1.14–5.86; *p* = 0.023; Fig. 2b) and multivariable (HR = 3.39; 95% CI 1.35–8.52; *p* = 0.0094; Supplementary Table 3) analyses.

**Table 3:** Survival analysis based on clinicopathological factors, including LAT1 protein expression, in ER-positive/HER2-negative patients.

Factors	Univariable analysis			Multivariable analysis		
	Hazard ratio	95% CI	*p*-value	Hazard ratio	95% CI	*p*-value
**LAT1 expression**						
Low	Reference			Reference		
High	2.86	1.26–6.48	0.012	2.97	1.16–7.62	0.023
**Ki67**						
< 10%	Reference			Reference		
≥ 10%	3.26	0.76–13.90	0.11	2.01	0.41–9.76	0.39
**PgR**						
Positive	Reference			Reference		
Negative	5.37	2.12–13.63	0.00041	4.62	1.80–11.82	0.0014
**Tumor size**						
pT1	Reference			Reference		
pT2-4	1.80	0.79–4.10	0.16	0.89	0.36–2.24	0.81
**Nodal status**						
Negative	Reference			Reference		
Positive	4.12	1.64–10.57	0.0027	4.32	1.58–11.79	0.0043
**Histological grade**						
Grade 1–2	Reference			Reference		
Grade 3	1.57	0.69–3.59	0.28	0.86	0.34–2.16	0.75

*LAT1* L-type amino acid transporter 1, *ER* estrogen receptor, *PgR* progesterone receptor, *HER2* human epidermal growth factor receptor 2.

## Discussion

The current study demonstrates that high LAT1 expression can be used to identify a subgroup of invasive BCa with aggressive behavior and high tumor immune reaction (PD-L1 positivity and TIL upregulation). The present study indicated that high LAT1 expression was an independent poor prognostic factor in luminal B-like BCa with long term follow-up.

The indications for chemotherapy in ER-positive/HER2-negative breast cancer patients have been debated for many years^29–31^. At the St. Gallen consensus conference^32^, Ki67 and PgR expression levels were suggested to be important factors to consider for postoperative chemotherapy. The St. Gallen consensus meeting recommended that hormone receptor-positive/HER2-negative breast cancer should be divided into the luminal A-like type (high ER/PgR and clearly low Ki67) and the luminal B-like type (low ER/PgR and clearly high Ki67)^32^. Adjuvant chemotherapy combined with endocrine treatment was recommended for luminal B-like type tumors. Recently, cyclin dependent kinase inhibitors have been suggested to be effective in ER-positive/HER2-negative BCa with a high risk of recurrence^33^. However, effective molecular targets for luminal B-like type tumors have not yet been fully identified as alternatives to chemotherapy. In this study, LAT1 expression was related to the expression of Myc and the VEGF family. VEGF and Myc may be related to proliferation in luminal B-like BCa^34^. LAT1 contributes to angiogenesis in cancer in the presence of VEGF^35^. Moreover, Myc asserts its oncogenic functions partially through its control of LAT1 expression^36^. The results of this study suggest that the molecular pathways involving LAT1 are related to the proliferation or metastasis abilities of luminal B-like BCa. It may be possible to support the development of new drugs targeted against luminal B-like BCa by conducting a more detailed functional analysis of the genes related to this molecular pathway.

El Ansari et al.^28^ clarified that glutamine transporters, including LAT1, are associated with the expression of CD68-positive macrophages and PD1-positive lymphocytes in tumors. Moreover, they used TNBC cell lines to demonstrate that the inhibition of LAT1 reduced the expression of PD-L1. HIF1α is known to activate tumor-associated CD68-positive macrophages^37^. Thus, LAT1 may be involved in the function of tumor-associated macrophages because it enhances the function of mTORC1 by controlling the HIF pathway^38^. The molecular pathway related to mTOR plays an essential role in the progression of BCa^39^. Although an mTOR inhibitor is used to treat metastatic ER-positive BCa in clinical practice^40^, its effectiveness in early-stage BCa remains to be elucidated. Recent investigations have observed an antitumor effect of using a LAT1 inhibitor because it suppresses the phosphoric acid of mTOR in tumor cells, inhibits its downstream cell proliferation signals, and elicits G1 arrest and apoptosis^41^. The mTOR pathway suppresses Treg cells and promotes the differentiation of CD8-positive T cells^42,43^. There are ongoing clinical trials for the combination of PI3K and PD-L1 inhibitors in TNBC^44^. To determine how LAT1 works in antitumor immune reactions, additional functional studies are necessary.

The present study revealed that high LAT1 expression was significantly associated with PD-L1 positivity at the protein level but not at the mRNA level. In recent years, with the rapid development and widespread use of comprehensive genomic analysis methods using microarrays and next-generation high-speed sequencing, candidate factors that may play a role in the prognosis and drug efficacy of BCa have been discovered from the analysis of vast amounts of genomic and transcriptomic information. However, although genomic and transcriptomic information is excellent for assessing the expression intensity of targets as continuous variables, it is difficult to determine how the factors are localized in the cells. The expression of PD-L1 in tumor cells is heterogeneous. It may bind not only to PD-L1-positive cancer cells but also to PD-L1-positive lymphocytes. In a recent study, PD-L1 expression on tumor cells was associated with high-risk clinicopathological parameters and poor prognosis, while PD-L1 expression on TILs was associated with favorable survival outcomes^45^. Further studies are needed to clarify the relationship between PD-L1 expression in tumor cells and lymphocytes and LAT1 expression in tumor cells.

In conclusion, LAT1 expression was associated with immune-related biomarkers, such as TILs and PD-L1, and was strongly correlated with poorly differentiated tumors. We evaluated TILs grade based on the guidelines of the International Working Group. Moreover, we examined the relationship of TILs and PD-L1 with LAT1 using the recent clinical breast cancer samples. These findings indicate that LAT1 may play important roles in antitumor immunity and may promote BCa progression and metastasis, particularly in ER-positive and HER2-negative BCa. This study has several limitations. First, the number of enrolled patients was small. Second, this study was a retrospective trial. Further biological research regarding the ability of this new agent to inhibit LAT1 expression is warranted^9^. Moreover, concomitant treatment using LAT1 inhibitors and immune checkpoint inhibitors^46^ is expected to become an innovative therapeutic target in the luminal B-like type of BCa.

## Materials and methods

### Patient characteristics

This study was ethically assessed by the Institutional Review Board of the Saitama Cancer Center (reference number 738). BCa patients (n = 250) who underwent breast surgery at the Saitama Cancer Center were included in this study. None of the patients included in this study received neoadjuvant treatment. In total, 199 (79.6%) patients underwent breast-conserving surgery, and 119 (47.6%) underwent axillary lymph node dissection. Of the 250 patients, 48.4% had pathological T2–4 tumors, and 45.2% were pathological lymph node metastasis-positive cases. All HER2-positive breast cancer patients did not receive adjuvant trastuzumab treatment, while patients with metastatic recurrent HER2-positive breast cancer received trastuzumab treatment after recurrence.

ER, PgR, HER2, and Ki67 labeling index were assessed as detailed in our previous studies^29,31^. The ER positivity (≥ 1%) rate was 67.6%; 58.0% of the patients were PgR-positive (≥ 20%), and 17.2% were HER2-positive. The cohort was classified according to the intrinsic molecular subtypes (luminal A-like, luminal B-like, HER2-positive, and triple-negative types). The luminal A-like type was defined as patients who are PgR-positive and display low Ki67 staining (labeling index of ≤ 10%) in ER-positive and HER2-negative breast cancer, whereas other ER-positive and HER2-negative tumors were classified as the luminal B-like type.

The density of stromal TILs was evaluated according to the International Working Group guidelines^24,47^. Cytoplasmic and/or membranous PD-L1 expression was assessed by immunohistochemistry (SP142; Roche, Switzerland; diluted 1:50), and the PD-L1 positivity cutoff value was determined to be 1%. Detailed assessments of these biomarkers have been described in our previous paper^25^.

### LAT1 immunohistochemistry

LAT1 expression was assessed by immunohistochemistry using an affinity-purified polyclonal rabbit anti-human LAT1 antibody^6^ diluted to 1:5000. LAT1 protein expression was evaluated for cytoplasmic and membrane-associated staining in full-face slides obtained from 250 patients. For clinicopathological and prognostic analyses, the 250 samples were stratified into high- and low-LAT1 expression groups based on staining intensity.

**Figure 3:**
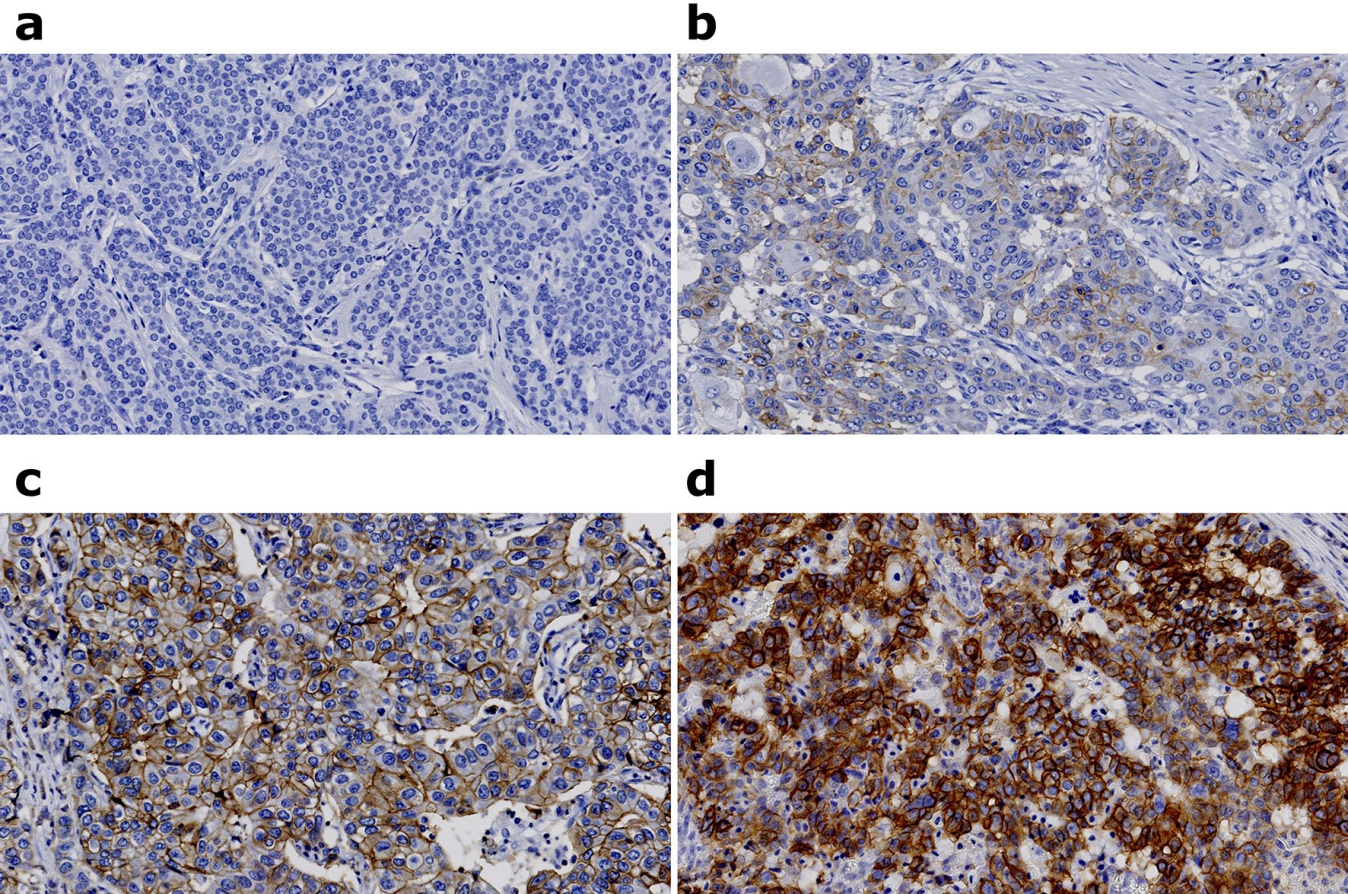
Immunohistochemical findings of L-type amino acid transporter 1 (LAT1) expression in breast cancer. (**a**) No staining (score 0), (**b**) weak staining (score 1), (**c**) moderate staining (score 2), and (**d**) strong staining (score 3) for LAT1 expression was detected in the cytoplasm of cancer cells.

The staining intensity of LAT1 expression on cancer cells was scored as follows: 0 (no staining or staining of < 10% of tumor cells), 1 (weak staining of ≥ 10% of tumor cells), 2 (moderate staining of ≥ 10% of tumor cells), and 3 (strong staining of ≥ 10% of tumor cells). In addition, tumors with a score of 2 or 3 were assigned to the high LAT1 expression group, whereas tumors with a score of 0 or 1 were assigned to the low LAT1 expression group (Fig. 3a-d: intensity score 0–3).

### Statistical analysis

Statistical analyses were performed using SPSS statistical software v24.0 (IBM, Armonk, NY, USA). The relationships between LAT1 expression and several clinicopathological factors were evaluated using the chi-square test and Fisher’s exact test. BCSS was used to evaluate the prognostic utility of LAT1 expression. For the univariable and multivariable prognostic assessments of several clinicopathologically important parameters (LAT1, tumor size, nodal status, histological grade, Ki67 labeling index, ER, PgR, HER2, and TILs), the Cox proportional hazards regression model was used to calculate HRs and 95% CIs. The prognostic value of LAT1 mRNA (*SLC7A5)* expression was further evaluated using the TCGA BRCA dataset as an external validation cohort. In brief, the datasets of mRNA expression from RNA-Seq V2 were accessed along with deidentified clinical information for several clinicopathological factors and outcomes^48,49^. The median value of *SLC7A5* expression was defined as the cutoff point.

### Ethical approval and informed consent

This study was approved by the Saitama Cancer Center Institutional Review Board (reference number 738). All procedures performed in studies involving human participants were conducted in accordance with the ethical standards of the institutional and/or national research committee and with the 1964 Declaration of Helsinki and its later amendments or comparable ethical standards. Informed consent was obtained from the participants included in this study.

## Supplementary Information

Supplementary Information 1.

Supplementary Information 2.

Supplementary Information 3.

Supplementary Information 4.

Supplementary Information 5.

Supplementary Information 6.

## Data Availability

The datasets generated and/or analyzed during the current study are not publicly available due to the regulation of the Institutional Review Board of the Saitama Cancer Center but are available from the corresponding author on reasonable request.
