# Supplementary figures and images for: Association of L-type amino acid transporter 1 (LAT1) with the immune system and prognosis in invasive breast cancer
Source: Sci Rep. 2022 Feb 17;12:2742. doi: 10.1038/s41598-022-06615-8 (PMC8854643; doi:10.1038/s41598-022-06615-8)

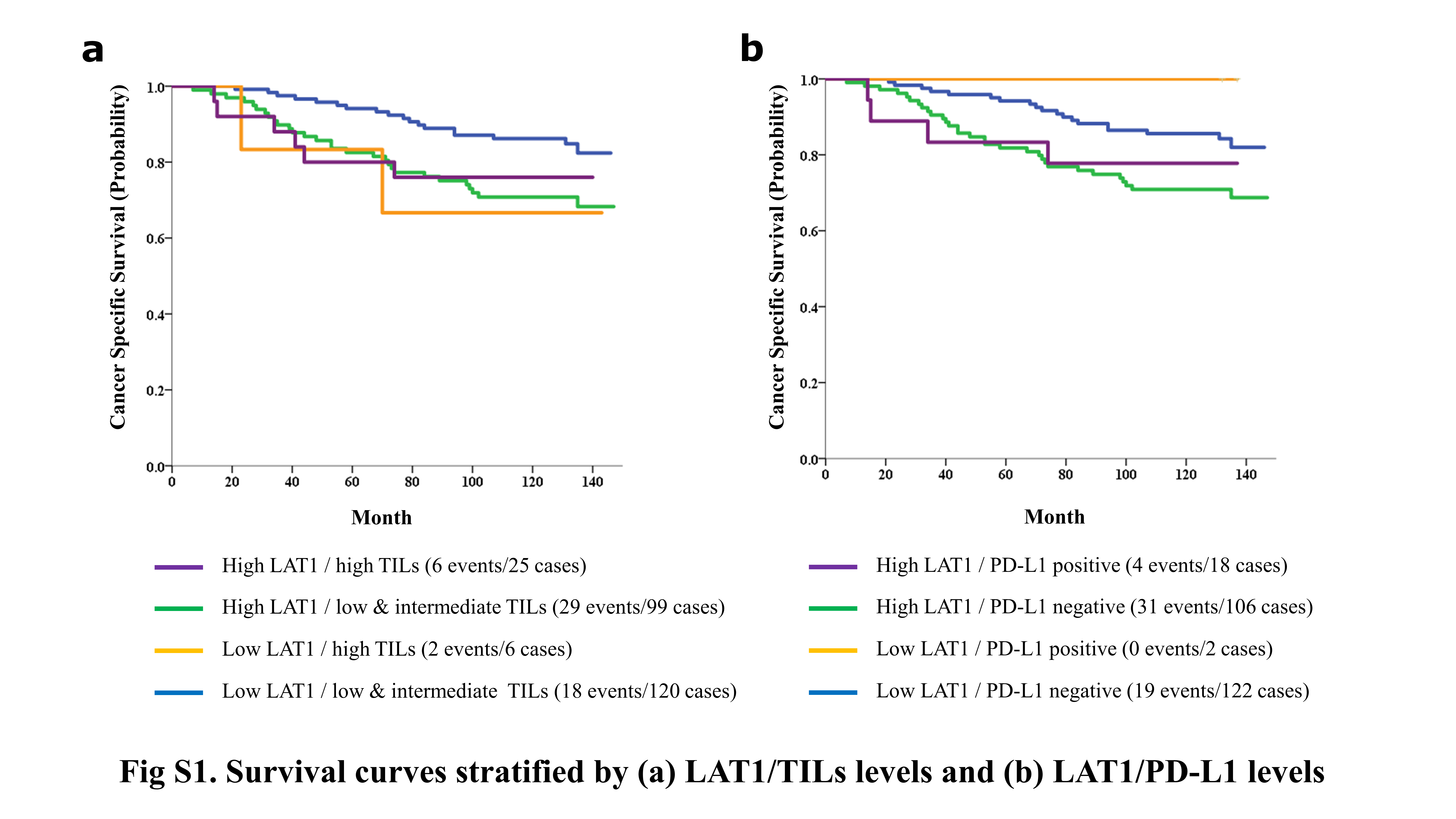

Supplement: Supplementary file 1 — Supplementary Information 1. [file 41598_2022_6615_MOESM1_ESM.tif]

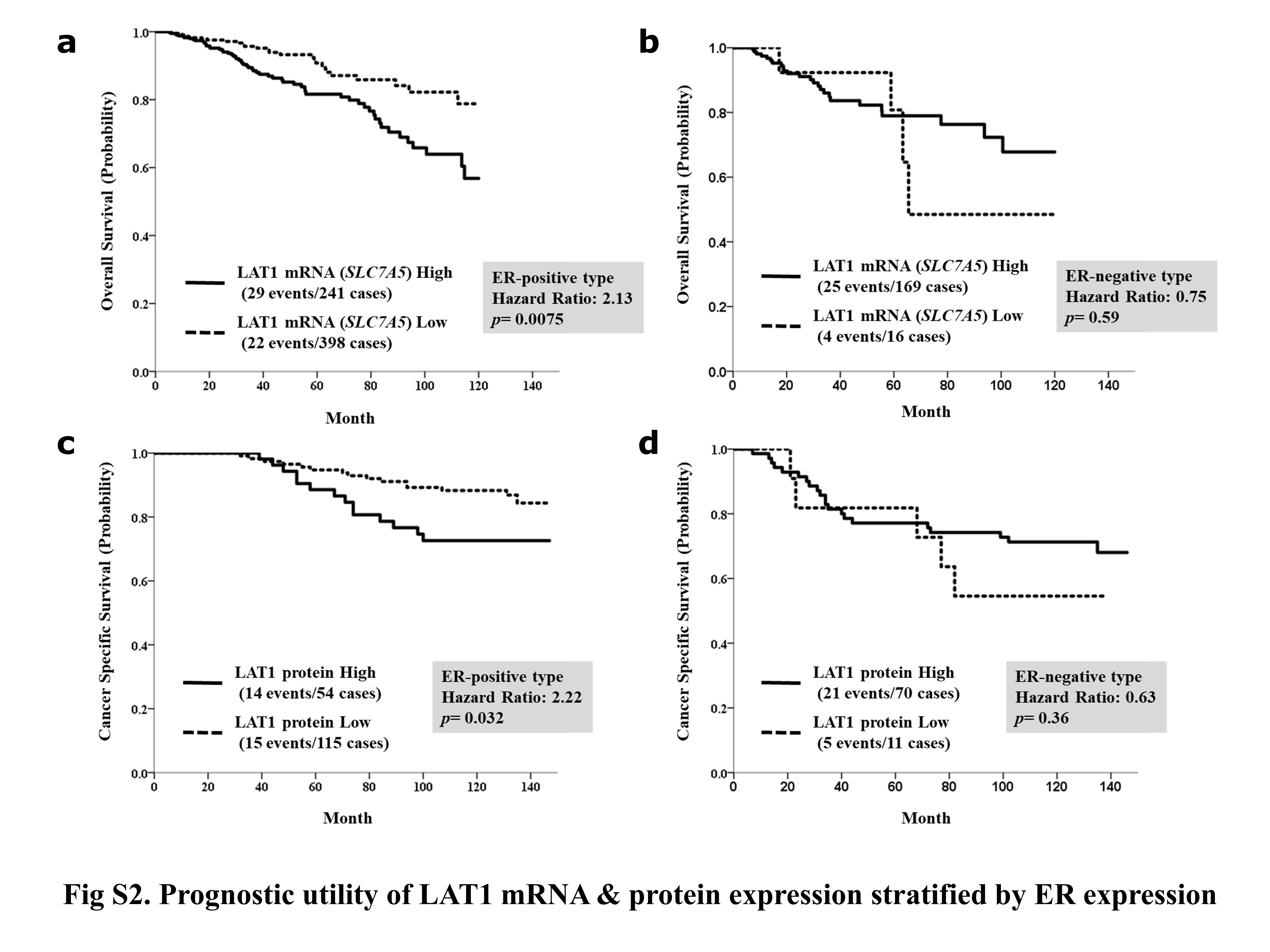

Supplement: Supplementary file 2 — Supplementary Information 2. [file 41598_2022_6615_MOESM2_ESM.tif]

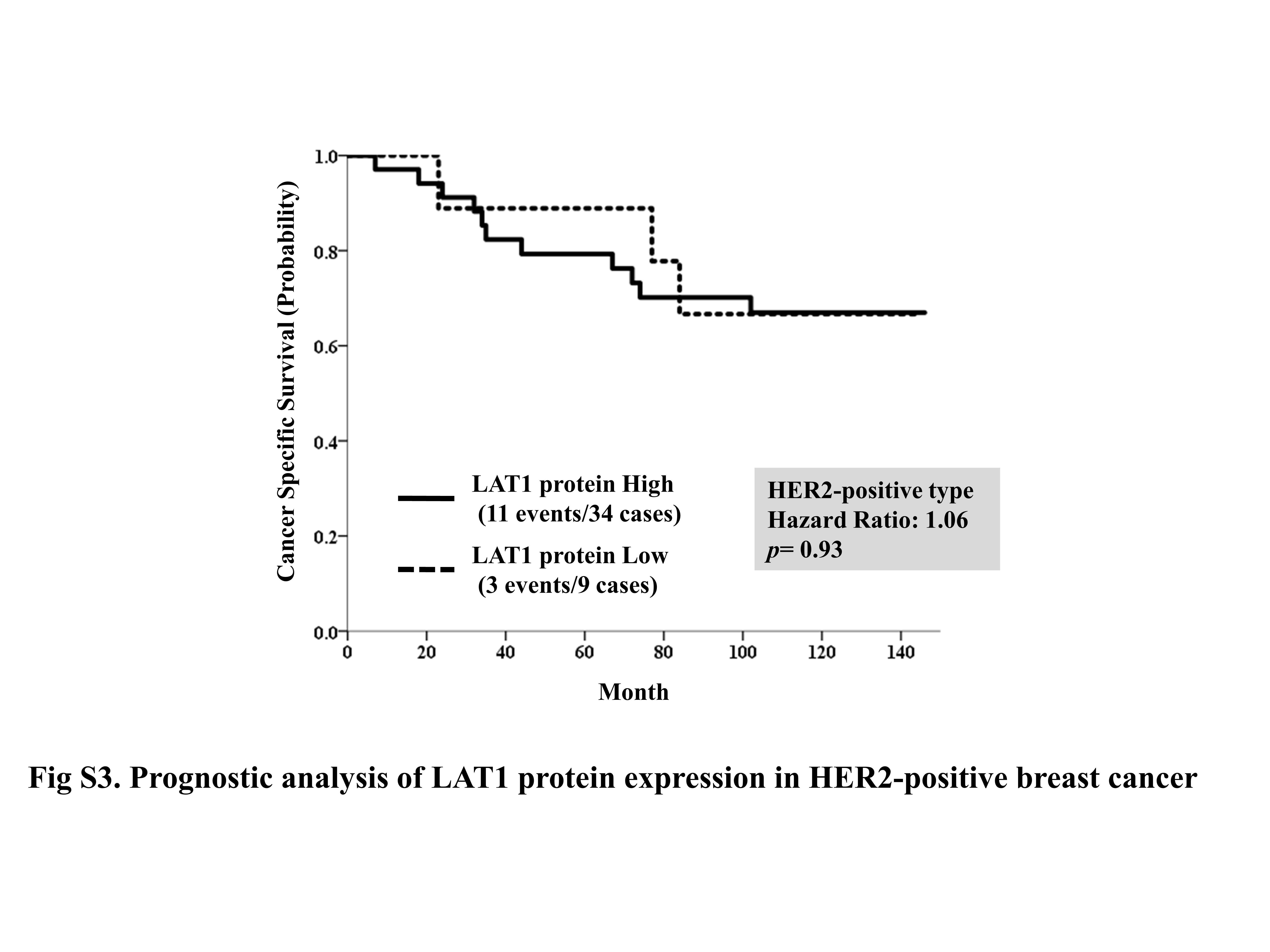

Supplement: Supplementary file 3 — Supplementary Information 3. [file 41598_2022_6615_MOESM3_ESM.tif]
