# Supplementary material for: Association of L-type amino acid transporter 1 (LAT1) with the immune system and prognosis in invasive breast cancer
Source: Sci Rep. 2022 Feb 17;12:2742. doi: 10.1038/s41598-022-06615-8 (PMC8854643; doi:10.1038/s41598-022-06615-8)
Supplement: Supplementary file 4 — Supplementary Information 4. [file 41598_2022_6615_MOESM4_ESM.docx]

**Supplementary Table 1** **Association of *LAT1*-mRNA expression with the clinicopathological factors**

| **Factors** | | **Expression of *LAT1*-mRNA** | | | **Significance** |
| --- | --- | --- | --- | --- | --- |
|  |  | **Low** | **High** | **Total** | ***p*-value** |
| **ER** | **Positive** | **398 (62.3%)** | **241 (37.7%)** | **639** | **< 0.0001** |
|  | **Negative** | **16 (8.6%)** | **169 (91.4%)** | **185** |  |
| **PgR** | **Positive** | **345 (63.2%)** | **201 (36.8%)** | **546** | **< 0.0001** |
|  | **Negative** | **64 (23.5%)** | **208 (76.5%)** | **272** |  |
| **HER2** | **Positive** | **57 (42.9%)** | **76 (57.1%)** | **133** | **0.067** |
|  | **Negative** | **295 (52.0%)** | **272 (48.0%)** | **567** |  |
| **Tumor size** | **pT2-4** | **294 (47.8%)** | **321 (52.2%)** | **615** | **0.047** |
|  | **pT1** | **133 (55.6%)** | **106 (44.4%)** | **239** |  |
| **Nodal status** | **Positive** | **213 (50.4%)** | **210 (49.6%)** | **423** | **0.78** |
|  | **Negative** | **210 (49.3%)** | **216 (50.7%)** | **426** |  |
| **Histological grade** | **Grade 3** | **78 (22.2%)** | **274 (77.8%)** | **352** | **< 0.0001** |
|  | **Grade 1, 2** | **322 (69.4%)** | **142 (30.6%)** | **464** |  |
| ***PD-L1* (*CD274*) mRNA** | **High** | **207 (47.7%)** | **227 (52.3%)** | **434** | **0.19** |
|  | **Low** | **220 (52.4%)** | **200 (47.6%)** | **420** |  |
| ***Myc1* mRNA** | **High** | **179 (41.9%)** | **248 (58.1%)** | **427** | **< 0.0001** |
|  | **Low** | **248 (58.1%)** | **179 (41.9%)** | **427** |  |
| ***VEGFA* mRNA** | **High** | **181 (42.4%)** | **246 (57.6%)** | **427** | **< 0.0001** |
|  | **Low** | **246 (57.6%)** | **181 (42.4%)** | **427** |  |
| ***VEGFB* mRNA** | **High** | **222 (52.0%)** | **205 (48.0%)** | **427** | **0.27** |
|  | **Low** | **205 (48.0%)** | **222 (52.0%)** | **427** |  |
| ***VEGFC* mRNA** | **High** | **244 (57.1%)** | **183 (42.9%)** | **427** | **< 0.0001** |
|  | **Low** | **183 (42.9%)** | **244 (57.1%)** | **427** |  |
| **Abbreviations: LAT1: L-type amino acid transporter 1, ER: estrogen receptor, PgR: progesterone receptor, HER2: human epidermal growth factor 2.** | | | | | |
