# Supplementary material for: Association of L-type amino acid transporter 1 (LAT1) with the immune system and prognosis in invasive breast cancer
Source: Sci Rep. 2022 Feb 17;12:2742. doi: 10.1038/s41598-022-06615-8 (PMC8854643; doi:10.1038/s41598-022-06615-8)
Supplement: Supplementary file 5 — Supplementary Information 5. [file 41598_2022_6615_MOESM5_ESM.docx]

**Supplementary Table 2 Survival analysis based on clinicopathological factors including protein expression of LAT1 in all patients.**

| **Factors** | | **Univariable analysis** | | | **Multivariable analysis** | | |
| --- | --- | --- | --- | --- | --- | --- | --- |
|  |  | **Hazard Ratio** | **95% CI** | ***p*-value** | **Hazard Ratio** | **95% CI** | ***p*-value** |
| **LAT1 expression** | **Negative** | **Reference** | | | **Reference** | | |
|  | **Positive** | **1.97** | **1.14-3.42** | **0.015** | **1.31** | **0.63-2.71** | **0.47** |
| **ER** | **Positive** | **Reference** | | | **Reference** | | |
|  | **Negative** | **2.13** | **1.26-3.62** | **0.0051** | **1.16** | **0.47-2.87** | **0.74** |
| **PgR** | **Positive** | **Reference** | | | **Reference** | | |
|  | **Negative** | **2.34** | **1.36-4.01** | **0.0021** | **1.91** | **0.85-4.26** | **0.12** |
| **HER2** | **Negative** | **Reference** | | | **Reference** | | |
|  | **Positive** | **1.80** | **0.98-3.30** | **0.058** | **1.08** | **0.55-2.11** | **0.82** |
| **Tumor size** | **< 2cm** | **Reference** | | | **Reference** | | |
|  | **> 2cm** | **2.05** | **1.18-3.55** | **0.011** | **1.68** | **095-2.97** | **0.074** |
| **Nodal status** | **Negative** | **Reference** | | | **Reference** | | |
|  | **Positive** | **3.80** | **2.10-6.87** | **< 0.0001** | **3.47** | **1.88-6.39** | **< 0.0001** |
| **Histological grade** | **Grade1-2** | **Reference** | | | **Reference** | | |
|  | **Grade 3** | **1.75** | **0.99-3.09** | **0.056** | **1.17** | **0.60-2.28** | **0.66** |
| **Tumor infiltrating lymphocytes** | **Low** | **Reference** | | | **Reference** | | |
|  | **Intermediate** | **0.81** | **0.38-1.74** | **0.60** | **1.61** | **0.71-3.64** | **0.25** |
|  | **High** | **0.69** | **0.26-1.85** | **0.46** | **1.14** | **0.41-3.17** | **0.80** |
