# Supplementary material for: Association of L-type amino acid transporter 1 (LAT1) with the immune system and prognosis in invasive breast cancer
Source: Sci Rep. 2022 Feb 17;12:2742. doi: 10.1038/s41598-022-06615-8 (PMC8854643; doi:10.1038/s41598-022-06615-8)
Supplement: Supplementary file 6 — Supplementary Information 6. [file 41598_2022_6615_MOESM6_ESM.docx]

**Supplementary Table 3 Survival analysis based on clinicopathological factors including protein expression of LAT1 in luminal B-like patients**

| **Factors** | | **Univariable analysis** | | | **Multivariable analysis** | | |
| --- | --- | --- | --- | --- | --- | --- | --- |
|  |  | **Hazard Ratio** | **95% CI** | ***p*-value** | **Hazard Ratio** | **95% CI** | ***p*-value** |
| **LAT1 expression** | **Low** | **Reference** | | | **Reference** | | |
|  | **High** | **2.58** | **1.14–5.86** | **0.023** | **3.39** | **1.35–8.52** | **0.0094** |
| **Tumor size** | **pT1** | **Reference** | | | **Reference** | | |
|  | **pT2-4** | **1.66** | **0.73–3.79** | **0.23** | **0.95** | **0.39–2.32** | **0.92** |
| **Nodal status** | **Negative** | **Reference** | | | **Reference** | | |
|  | **Positive** | **3.99** | **1.57–10.12** | **0.0036** | **4.82** | **1.83–12.72** | **0.0015** |
| **Histological grade** | **Grade1-2** | **Reference** | | | **Reference** | | |
|  | **Grade 3** | **1.27** | **0.56–2.90** | **0.57** | **0.85** | **0.35–2.09** | **0.72** |
